# SUMOylation of Kir7.1 participates in neuropathic pain through regulating its membrane expression in spinal cord neurons

**DOI:** 10.1111/cns.13871

**Published:** 2022-05-27

**Authors:** You‐You Lv, Han Wang, Hai‐Ting Fan, Ting Xu, Wen‐Jun Xin, Rui‐Xian Guo

**Affiliations:** ^1^ Department of Anesthesiology, The Fifth Affiliated Hospital Sun Yat‐Sen University Zhuhai China; ^2^ Department of Anesthesiology, Changhai Hospital Naval Medical University Shanghai China; ^3^ Department of Physiology and Pain Research Center, Zhongshan School of Medicine Sun Yat‐Sen University Guangzhou China

**Keywords:** Kir7.1, neuropathic pain, spinal cord, SUMOylation

## Abstract

**Aims:**

Potassium (K^+^) channels have been demonstrated to play a prominent involvement in nociceptive processing. Kir7.1, the newest members of the Kir channel family, has not been extensively studied in the CNS, and its function remains largely unknown. The present study investigated the role of spinal Kir7.1 in the development of pathological pain.

**Methods and Results:**

Neuropathic pain was induced by spared nerve injury (SNI). The mechanical sensitivity was assessed by von Frey test. Immunofluorescence staining assay revealed that Kir7.1 was predominantly expressed in spinal neurons but not astrocytes or microglia in normal rats. Western blot results showed that SNI markedly decreased the total and membrane expression of Kir7.1 in the spinal dorsal horn accompanied by mechanical hypersensitivity. Blocking Kir7.1 with the specific antagonist ML418 or knockdown kir7.1 by siRNA led to mechanical allodynia. Co‐IP results showed that the spinal kir7.1 channels were decorated by SUMO‐1 but not SUMO‐2/3, and Kir7.1 SUMOylation was upregulated following SNI. Moreover, inhibited SUMOylation by GA (E1 inhibitor) or 2‐D08 (UBC9 inhibitor) can increase the spinal surface Kir7.1 expression.

**Conclusion:**

SUMOylation of the Kir7.1 in the spinal cord might contribute to the development of SNI‐induced mechanical allodynia by decreasing the Kir7.1 surface expression in rats.

## Graphical abstract

SNI markedly decreased the expression of Kir7.1 and upregulated SUMOylated‐Kir7.1 in the spinal dorsal horn accompanied by mechanical hypersensitivity. Blocking Kir7.1 with the specific antagonist or knockdown kir7.1 by siRNA led to mechanical allodynia. Moreover, inhibited SUMOylation can increase the spinal surface Kir7.1 expression. SUMOylation of the Kir7.1 in spinal cord might contribute to the development of SNI‐induced mechanical allodynia by decreasing the Kir7.1 surface expression in rats.

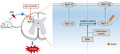

## 1 INTRODUCTION

Neuropathic pain is a pathological condition resulting from a lesion or disease of the somatosensory nervous system and which affects 7% ~ 10% of the general population.^1^ It is characterized by abnormal pain responses, burning sensation, or numbness, etc.^2^, ^3^ Furthermore, chronic neuropathic pain may lead patients to suffer from poor life experiences such as sleep disturbances, anxiety, or depression and which impaired the quality of life severely.^4^, ^5^ Many efforts have been made to reveal the etiology of neuropathic pain, while the underlying mechanism of the neuropathic pain is still largely unclear, and effective treatment for chronic pain is still a huge unmet in the clinic.

Potassium (K^+^) channels play an essential role in establishing the resting membrane potential and are crucial determinants of neuronal activity throughout the nervous system.^6^ Dysfunction of K^+^ channels is linked to many kinds of human diseases or disorders related to nervous system, including neuropathic pain.^7^, ^8^, ^9^ On the basis of structural and physiological attributes, K^+^ channels are classified mainly into voltage‐gated (K_v_), two‐pore (K_2P_), calcium‐activated (K_CA_), and inward‐rectifying (Kir) channels.^10^ Kir7.1 is the most recently described Kir subtype.^11^ Recent study has shown that Kir7.1 is expressed pervasive in the brain than previously recognized and have potential importance in regulating neuronal and glial function.^12^ Evidence have shown that some K^+^ channels (for example Kv1,^13^ Kv2,^14^ Kv7^15^ and TREK2^16^) are involved in the development and maintenance of neuropathic pain; however, the role of Kir7.1 in neuropathic pain remains largely unexplored. In the present study, we investigated the role of Kir7.1 in SNI‐induced mechanical allodynia.

SUMOylation is a post‐translational modification in which a member of the small ubiquitin‐like modifier (SUMO) family of proteins is conjugated to lysine residues in target proteins.^17^ Such a modification can facilitate or prevent inter‐ and intra‐molecular interactions via conformational changes or direct steric hindrance.^18^ In mammals, there are three SUMO paralogues (SUMO1‐3).^19^ The three SUMO proteins can be covalently conjugated to proteins as a single moiety (SUMO‐1) or as polymeric SUMO chains (SUMO‐2/3).^20^ Similar to ubiquitylation, SUMOylation is regulated by a specialized set of activating (E1), conjugating (E2), and ligating (E3) enzymes, and is reversed by specific isopeptidases referred to as sentrin/SUMO‐specific proteases (SENPs).^21^

Emerging evidence established that SUMOylation of proteins plays key roles in neuronal function. An increasing number of ion channels, including Kainite receptor GluR6,^22^ Kv2.1,^23^ Kv1.5,^24^ and transient receptor potential melastatin ion channel type 4 (TRPM4),^24^ transient receptor potential vanilloid 1 (TRPV1)^18^ and hyperpolarization‐activated cyclic nucleotide‐gated ion channel 2 (HCN2),^25^ have been reported to be conjugated and regulated by SUMO, suggesting that SUMOylation might be a common mechanism for modulating ion channel function.^18^, ^26^ Here, we investigated whether Kir7.1 was modified by SUMOylation in SNI rats.

## 2 MATERIALS AND METHODS

### 2.1 Animals

Male Sprague–Dawley rats (200–220 g, purchased from the Institute of Experimental Animals of Sun Yat‐sen University) were group‐housed with ad libitum access to food and water in a temperature (24°C) and humidity (50%–60%) and light‐controlled (12:12‐h light/dark cycle) quiet room. All experimental procedures were approved by the local Animal Care and Use Committee (No. SYXK [yue] 2015–0107) and were performed in accordance with the guidelines of the National Institutes of Health on animal care and the ethical guidelines.

### 2.2 Spared nerve injury

Spared nerve injury (SNI) surgery was performed as previously described.^27^ Briefly, rats were anesthetized with sodium pentobarbital (50 mg/kg, Sigma‐Aldrich) intraperitoneally (ip.). The left sciatic nerve and its three terminal branches (the sural, common peroneal, and tibial nerves) were exposed through an incision at the midthigh level. The common peroneal and tibial nerves were tightly ligated with 5.0 silk and sectioned (removal of a 2 mm length). The sural nerve was kept intact. In the sham group, identical operation was performed for exposure of the sciatic nerve and its branches but without any nerve injury.

### 2.3 Behavioral tests

Mechanical withdrawal thresholds of the rats were assessed by the up‐down method as described previously.^28^, ^29^ Briefly, rats were acclimatized to the testing environment for 3 consecutive days (2 h/d). After habituation for 15 min, a series of von Frey hairs of bending force (0.41, 0.70, 1.20, 2.04, 3.63, 5.50, 8.51, and 15.14 g) were applied to the plantar surface of the hind paw, which is predominantly innervated by the sciatic nerve. The brisk withdrawal or licking of the paw in response to the stimulus was considered as a positive response. The 50% paw withdrawal thresholds (PWT) were calculated.

### 2.4 Intrathecal catheter implantation

For intrathecal (i.t.) delivery of drugs, rats were implanted with i.t. catheters as described previously.^30^ Briefly, after being anesthetized, a sterile polyethylene (PE‐10) tube was inserted through L5/L6 intervertebral space and the tip of the tube was placed at the spinal lumbar enlargement level. Any rats that developed hind limb paralysis or paresis after surgery were excluded.

### 2.5 Drug administration

Ginkgolic acid (GA, a potent sumoylation inhibitor) and 2‐D08 (an UBC9 inhibitor) were purchased from Selleck Chemicals. GA (10, 50, 100, or 200 μM) or 2‐D08 (10, 30, 60, or 100 μM) or vehicle (0.2% DMSO) was i.t. injected from Day 11 to Day 15 once daily after SNI. The drugs were i.t. injected in a volume of 10 μl followed by additional 10 μl of vehicle to flush the catheter.

### 2.6 Western blot

Western blot was performed following our previous study.^30^ After being anesthetized with sodium pentobarbital (50 mg/kg, i.p.), the L_4‐6_ spinal dorsal horn segments were immediately removed and homogenized in ice‐cold lysis buffer [RIPA lysis buffer (Beyotime Institute of Biotechnology) for total protein, Plasma Membrane Protein Extraction Kit (Invent Biotechnologies Inc.) for membrane protein] containing a cocktail of proteinase inhibitors (Roche). The protein samples were separated by SDS‐PAGE and electrotransfered onto PVDF membranes. The membranes were incubated with the primary antibody against Kir7.1 (1:500, Alomone), SUMO1 (1:1000, CST), UBC9 (1:1000, abcam), SUMO2/3 (1:1000, CST), β‐actin (1:1000, CST), or TfR (1:500, Invitrogen) over night at 4°C and then with HRP‐conjugated secondary antibody for 1 h at room temperature (RT). The immune complex was visualized using enhanced chemiluminescence (Thermo Scientific) and quantified by NIH ImageJ.

### 2.7 Immunohistochemistry

Immunochemistry was performed in accordance with the previous description.^31^ In short, perfusion was performed through the ascending aorta with 4% paraformaldehyde under deeply anesthetized. The lumber spinal cord was removed, post‐fixed in the same fixative for 2 h, and then transferred into 30% sucrose until sectioning. Cryostat transverse spinal sections (15 μm) were cut and processed for immunohistochemistry with primary antibody for Kir7.1 (1:200, Alomone), SUMO1 (1:100, CST), NeuN (1:400, Millipore), GFAP (1:400, CST), and Iba1 (1:200, Abcam) overnight at 4°C. Then, the sections were incubated with the Alex 568‐ or Alex 488‐conjugated secondary antibodies for 1 h at RT. The stained sections were examined with NIKCON fluorescence microscope, and images were captured with a NIKCON camera. Nonspecific staining was determined by omitting the primary antibodies.

### 2.8 Co‐Immunoprecipitation assay

Co‐Immunoprecipitation (Co‐IP) was carried out following our previous study.^29^ Briefly, the extracted spinal cord tissues were lysed in IP‐RIPA buffer (Beyotime Institute of Biotechnology, China) with protease inhibitors and N‐ethylmaleimide (NEM, 20 mM) with rotation at 4°C for 30 min. After centrifugation (10 min at 12,000 rpm), the supernatant was transferred to fresh tubes. The SUMO1 antibody (1:50) or Kir7.1 antibody, which immobilized with resin, were used to collect the immune complexes. The eluted complexes from the resin were analyzed by Western blot using Kir7.1 antibody or SUMO1 antibody after incubation and washes.

### 2.9 SiRNA preparation and transfection

Specific siRNAs were applied to knockdown the expression of Kir7.1 as previously described.^29^ Briefly, siRNA targeting rat KCNJ13 (Kir7.1 encoding‐gene) were designed and synthesized by Ribobio Biotechnology, the siRNA with the nucleotide sequences is 5′‐ UCUUUGUGAGCUACAACUGCU ‐3′ (sense) and 5′‐ CAGUUGUAGCUCACAAAGAUG −3′ (antisense). Kir7.1 siRNA (1 nmol, 10 μl) were i.t. injected once daily for 10 consecutive days. The control group received the same volume of scramble siRNA.

### 2.10 Statistical analysis

All data were presented as means ± standard deviation (SD) and analyzed with SPSS 20.0 software. Western blot and qPCR data were analyzed by two‐tailed, independent Student's t‐test and two‐way ANOVA followed by a Tukey post hoc test. Behavioral results were performed by one‐way or two‐way ANOVA with repeated measures followed by a Tukey post hoc test. *p* < 0.05 was considered statistically significant.

## 3 RESULTS

### 3.1 The expression of Kir7.1 in the neuron of spinal cord dorsal horn was downregulated in SNI rats

**FIGURE 1:**
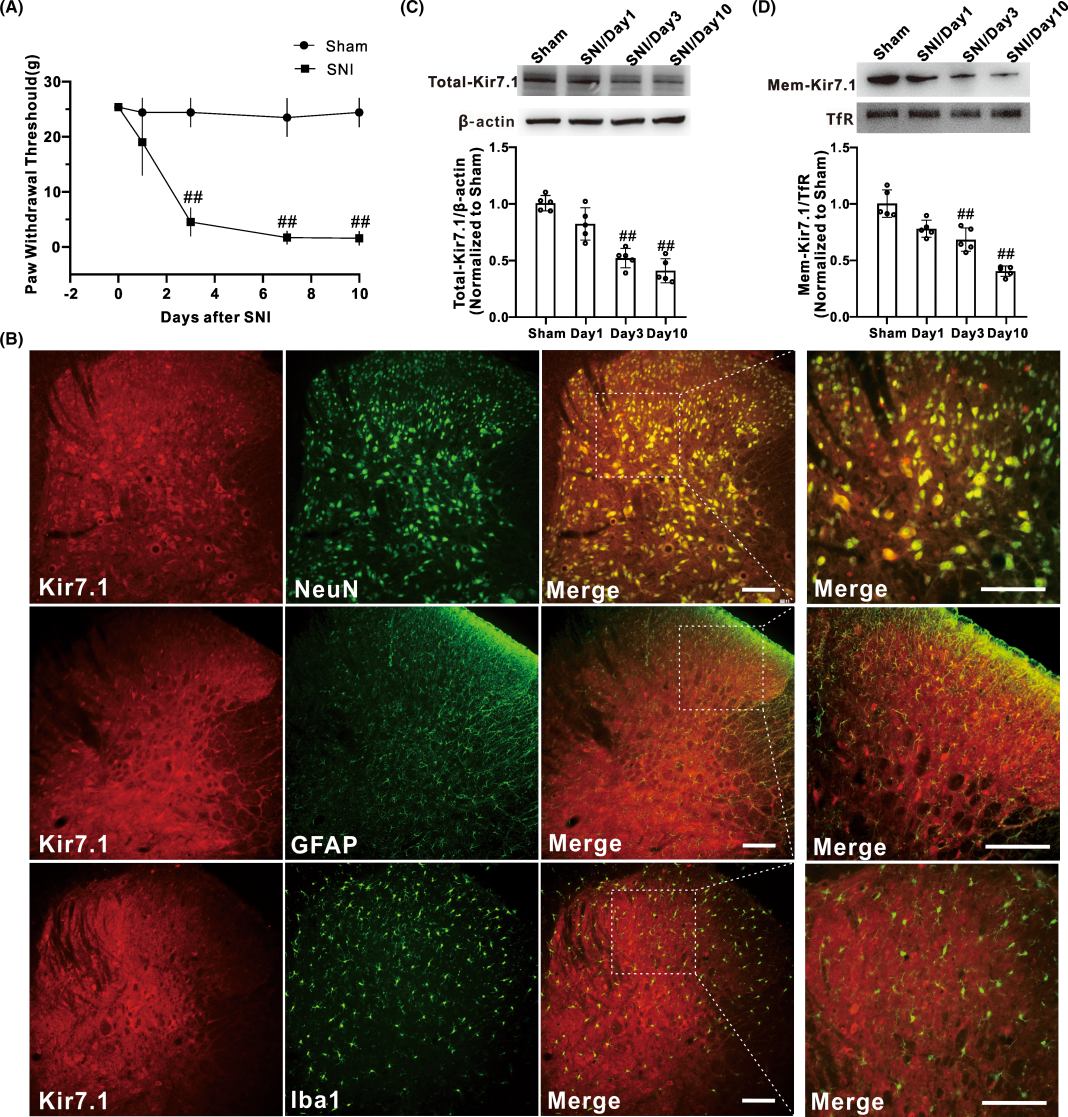
Membrane expression of Kir7.1 was downregulated after SNI treatment. (A) Hind paw withdrawal threshold response to von Frey filament stimuli were measured, and SNI induced significant mechanical allodynia, which started on Day 3 and lasted for at least 10 days (*n* = 8 in each group, ^##^ *p* < 0.01 vs. Sham). (B) Immunofluorescence staining assessing the colocalization of Kir7.1 (red) with the cell‐specific markers: NeuN (neuronal marker, green), GFAP (astrocyte marker, green), and Iba1 (microglia marker, green) in spinal cord tissues in normal rats, *n* = 3 rats, Scale bar, 100 μm. (C) Representative histogram and blots showed that the total expression of Kir7.1 in protein level was downregulated on Day 3 and Day 10 after SNI (*n* = 5 in each group, ^##^ *p* < 0.01 vs sham). (D) Representative histogram and blots showed the downregulation of membrane Kir7.1 protein on Day 3 and Day 10 after SNI (*n* = 5 in each group, ^#^ *p* < 0.05 vs. sham, ^##^ *p* < 0.01 vs. sham)

To evaluate the development of mechanical allodynia, PWTs were recorded at baseline (Day 0) and Days 1, 3, 7, and 10 after SNI surgery in rats. Consistent with earlier findings, we confirmed that unilateral SNI induced a long‐lasting decrease in ipsilateral PWTs (*P* < 0.05) from Day 3 to Day 10 in SNI rats compared with the sham group (Figure 1A).

To explore the role of Kir7.1 in the SNI‐induced mechanical allodynia, we first examined the expression and cellular location of Kir7.1 in the spinal cord. Double immunofluorescence staining experiments showed that Kir7.1 was predominantly co‐localized with NeuN (a marker of neuron), but not with GFAP (a marker of astrocyte) or Iba1 (a marker of microglia) (Figure 1B), suggesting that Kir7.1 was expressed in spinal neurons but not astrocytes or microglia in normal rats. Western blot results showed that the total expression of Kir7.1 in the spinal dorsal horn of the SNI group was decreased significantly compared with the sham group, which exhibited a time course similar to that of the increase in the mechanical hypersensitivity in rats after SNI (Figure 1C). As an ion channel, Kir7.1, only when it is located on the cell membrane, is functional. Thus, we also detected the membrane expression of Kir7.1 in the spinal dorsal horn. The results showed that the membrane Kir7.1 was significantly downregulated after SNI (Figure 1D). These data indicated that SNI downregulated the expression of Kir7.1, especially the membrane Kir7.1 in the spinal dorsal horn. Kir7.1 may be involved in the development of SNI‐induced chronic pain.

### 3.2 Blocking Kir7.1 channel or knockdown Kir7.1 induced the mechanical allodynia

**FIGURE 2:**
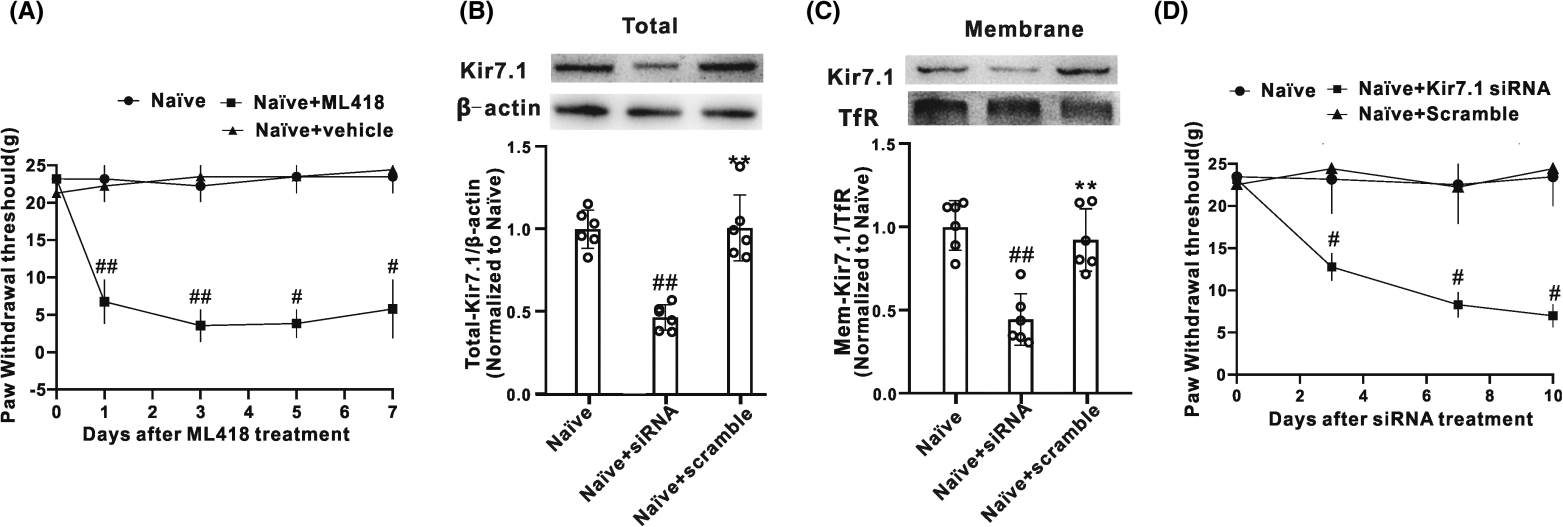
Kir7.1 contributed to the development of mechanical allodynia. (A) intraperitoneal application of ML418 (a first select Kir7.1 channel blocker, 30 mg/kg, 10 μl) for 5 consecutive days produced significant mechanical allodynia, which started on Day 1 and lasted for at least 7 days (*n* = 4 in each group, ^#^ *p* < 0.05 vs. the Naïve+vehicle group, ^##^ *p* < 0.01 vs. the Naïve+vehicle group). (B and C) The expression of both total Kir7.1 and membrane Kir7.1 in spinal cord were suppressed on Day 10 following consecutive intrathecal injection of Kir7.1 siRNA for 10 days (*n* = 6 in each group, ^##^ *p* < 0.01 vs. the Naïve+scramble group; siRNA, Kir7.1 siRNA; scramble, scramble siRNA). (D) Intrathecal administration of Kir7.1 siRNA (1 nmol, 10 μl) for 10 consecutive days significantly induced mechanical allodynia (*n* = 4 in each group, ^#^ *p* < 0.05 vs. the Naïve+scramble group)

To investigate whether inhibition of the Kir7.1 channel would affect the mechanical sensitivity, ML418 (30 mg/kg, i.p.), the first selective blocker of Kir7.1 channel^32^ was administered for consecutive 5 days. As shown in Figure 2A, the PWTs were significantly decreased in the ML418‐treated group as compared to the naïve or vehicle group. Furthermore, we observed the effect of Kir7.1 knockdown in dorsal horn on pain sensitivity by Kir7.1 siRNA (1 nmol/10 μl, i.t.) for 10 consecutive days in naive rats. As shown in Figure 2B and C, both the total and the membrane protein levels of Kir7.1 were significantly inhibited in the siRNA group, indicating the efficiency of Kir7.1 siRNA. Meanwhile, the PWT in response to mechanical stimuli was decreased obviously in the siRNA group (Figure 2D), indicating that the RNAi‐mediated deletion of Kir7.1 dramatically induced the mechanical allodynia. These results suggested that downregulation of spinal Kir7.1 mediated the chronic pain induced by SNI.

### 3.3 SUMO1‐mediated SUMOylation of Kir7.1 was significantly upregulated in the spinal cord after SNI

**FIGURE 3:**
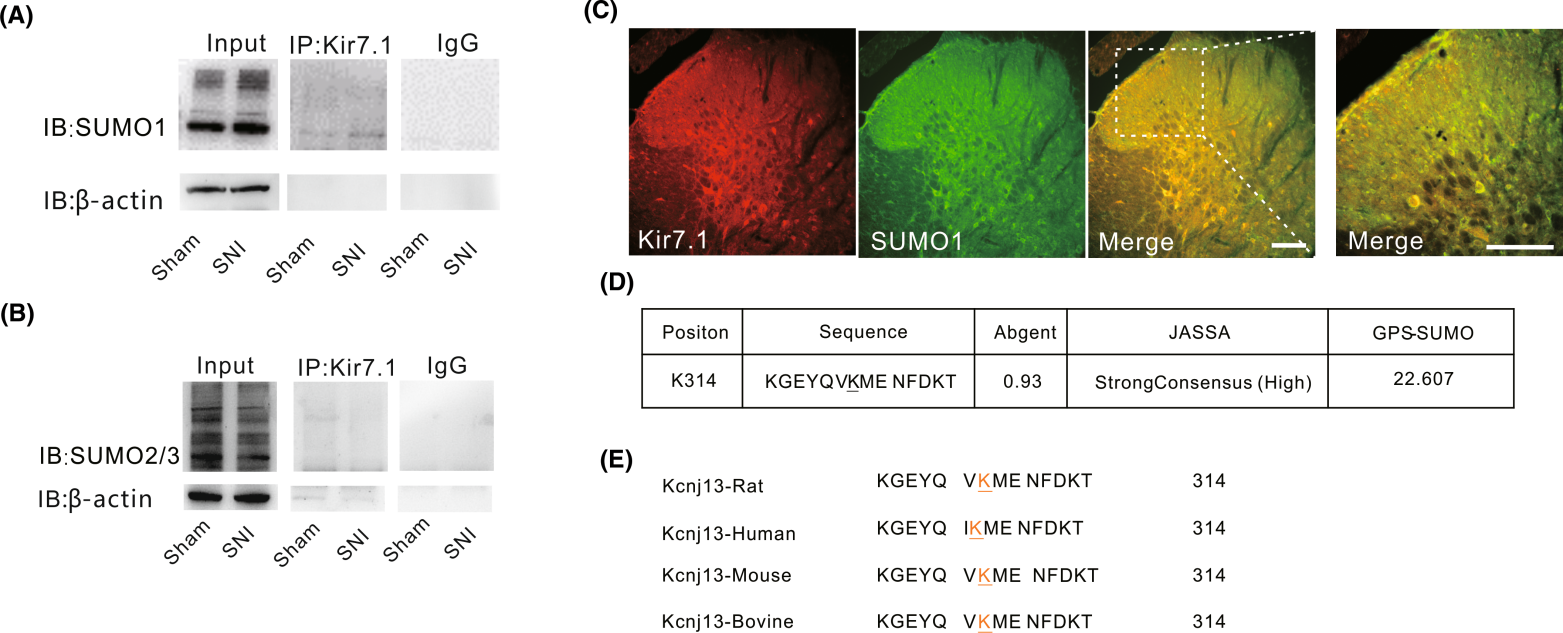
SUMO1‐mediated SUMOylation of Kir7.1 was upregulated in the spinal cord after SNI. (A) Upreguation in SUMO1 was immunoprecipitated with Kir7.1 antibody on Day 10 after SNI (*n* = 6 rats per group. IgG, immunoglobulin G; IB, immunoblot). (B) Co‐immunoprecipitation (IP) of SUMO2/3 with antibody to Kir7.1 in lysates from spinal cord tissues isolated from rats at 10 days after SNI or the sham group (*n* = 6 rats per group). (C) Immunohistochemistry assessing the colocalization of Kir7.1 with SUMO1 in spinal cord tissues in normal rats (*n* = 3 rats, Scale bar, 100 μm). (D) Details of the SUMO‐conjugation motifs and summary of prediction software scores. (E) Alignment of Kir7.1 sequences from different species as indicated at the consensus SUMOylation site

Previous studies have reported that several ion channels can be SUMOylated, suggesting that SUMOylation might be a common mechanism for modulating ion channel function. SUMOylation of Kir7.1 has never been examined. To assess whether Kir7.1 can be regulated by SUMOylation in SNI‐induced chronic pain, Co‐IP was performed with SUMO1‐ or SUMO2/3‐specific antibody. The results showed that the potential binding of SUMO1 with Kir7.1, but not SUMO2/3, was increased at Day 10 after SNI (Figure 3A, B), indicating that Kir7.1 could be post‐translationally modified by SUMO1 in the rat spinal cord. In addition, immunofluorescence results showed that Kir7.1 co‐localized with SUMO1 in the spinal cord (Figure 3C). These results implied that Kir7.1 SUMOylation may be dynamically regulated by SNI.

SUMO‐modified proteins typically contain a consensus tetrapeptide motif which allow proteins to bind SUMO proteins non‐covalently.^17^ Thus, we performed bioinformatics analysis to detect SUMOylation sites of rat Kir7.1 protein sequence using three independent computational programs (SUMOplotTM Analysis Programme [Abgent], JASSA, and GPS‐SUMO). The results identified one lysine residues at position 314 adhere to the consensus (Figure 3D). Then, we verified the conservation of this motif in different organisms. It is intriguing that K314 is conserved among mammals (e.g., rat, human, mouse, and bovine) (Figure 3E). Taken together, we proposed that K314 might be the potential SUMOylated modification site of Kir7.1 in rat.

### 3.4 SNI‐induced upregulation of SUMOylated Kir7.1 was prevented by Ginkgolic acid and 2‐D08

**FIGURE 4:**
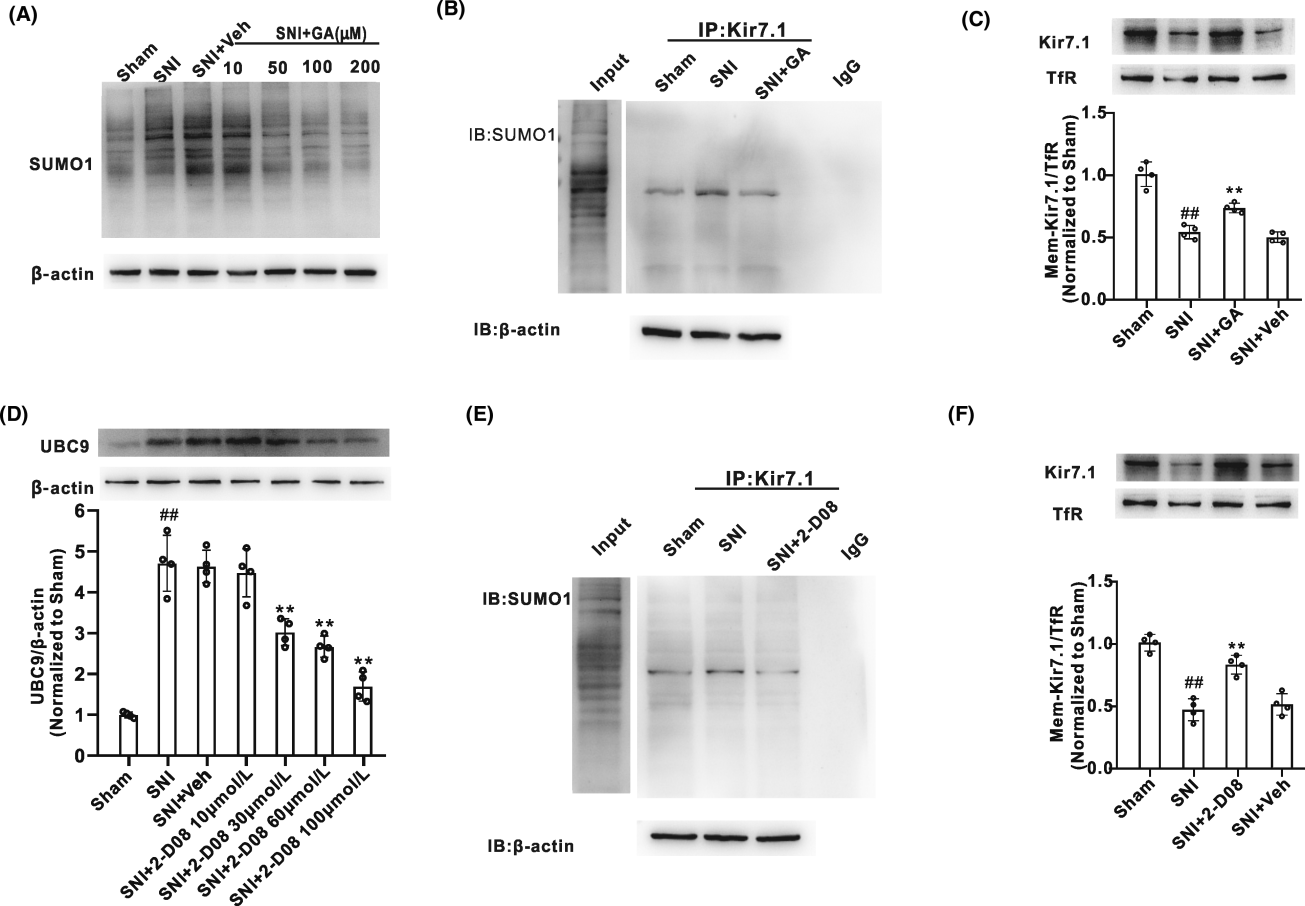
Inhibition of Kir7.1 SUMOylation reversed the downregulation of membrane Kir7.1 expression in the development of neuropathic pain induced by SNI. (A) Abundance of SUMO1 protein in spinal cord horn of rats, which were injected ginkgolic acid (GA) intrathecally at different concentration (10, 50, 100, or 200 μM) for 5 consecutive days since 11 days after SNI treatment (*n* = 4 in each group). (B) Co‐IP of SUMO1 with antibody to Kir7.1 in lysates from spinal cord tissues isolated from rats treated with Sham, SNI, or SNI + GA (100 μM) (*n* = 4 rats per group). (C) Abundance of membrane expression of Kir7.1 in spinal cord induced by GA treatment for 5 consecutive days from Day 11 after SNI (*n* = 4 in each group, ^##^ *p* < 0.01 vs. the Sham group, ^**^ *p* < 0.01 vs. the SNI + Veh). (D) Western blot analysis of UBC9 protein expression in spinal cord horn of rats, which were injected 2‐D08 intrathecally at different concentration (10, 30, 60, or 100 μM) for 5 consecutive days since 11 days after SNI treatment (*n* = 4 in each group, ^##^ *p* < 0.01 vs. the sham group, ^**^ *p* < 0.01 vs. the SNI + Veh group; Veh, vehicle). (E) Co‐IP of SUMO1 with antibody to Kir7.1 in lysates from spinal cord tissues isolated from rats treated with Sham, SNI, or SNI + 2‐D08 (60 μM) (*n* = 4 rats per group). (F) Intrathecal injection of 2‐D08 (60 μM) for 5 consecutive days from Day 11 after SNI treatment induced the increase in membrane expression of Kir7.1 in spinal cord, compared with the SNI + Veh group (*n* = 4 in each group, ^##^ *p* < 0.01 vs. the Sham group, ^**^ *p* < 0.01 vs. the SNI + Veh)

SUMOylation is mediated by an enzymatic cascade reaction and SUMO conjugation is achieved by different enzymes, E1‐activating, E2‐conjugating, and various E3 ligases.^21^ GA, an E1 inhibitor, which could inhibit protein SUMOylation by blocking the formation of the E1‐SUMO intermediate, was administered (10, 50, 100, or 200 μM, i.t.) for 5 consecutive days from Day 11 after SNI surgery. As Figure 4A showed, SNI markedly increased the level of SUMO1‐conjugated proteins in spinal cord. GA at the concentration of 100 μM could effectively inhibit the increase in protein SUMOylation induced by SNI. Thus, we chose GA at the concentration of 100 μM in the subsequent experiments. Co‐IP results showed that GA (100 μM, i.t.) effectively inhibited the Kir7.1 SUMOylation in the spinal cord after SNI (Figure 4B). Additionally, Western blot results showed that GA treated reversed the downregulation of membrane Kir7.1 expression on Day 10 after SNI (Figure 4C). Furthermore, 2‐D08, a select UBC9 inhibitor, was administrated i.t. for 5 consecutive days from 11 days after SNI. 2‐D08 treatment significantly decreased the upregulation of UBC9 protein (Figure 4D) and SUMOylated Kir7.1 (Figure 4E) in the spinal cord induced by SNI. Moreover, we observed that 2‐D08 increased the surface accumulation of Kir7.1 in the spinal cord at 15 days after SNI (Figure 4F). Taken together, these results indicated that SUMOylation upregulated the surface Kir7.1 expression after SNI.

## 4 DISCUSSION

In the present study, we showed that Kir7.1 expression in the spinal cord neurons was decreased following SNI. Blocking Kir7.1 with the specific antagonist ML418 or knockdown Kir7.1 by siRNA led to mechanical hypersensitivity. These results indicate that downregulation of Kir7.1 might contribute to the development of SNI‐induced mechanical allodynia. Furthermore, we demonstrated that kir7.1 channels were decorated by SUMO‐1 but not SUMO‐2/3 in the dorsal horn, suggesting that the spinal kir7.1 can be SUMOylated in rats. Moreover, Kir7.1 SUMOylation was upregulated in SNI rats. Inhibited SUMOylation by GA (an E1 inhibitor) or 2‐D08 (UBC9 inhibitor) can increase the spinal membrane kir7.1. Taken together, these findings suggest that SUMOylation of Kir7.1 may play a critical role in SNI‐induced mechanical allodynia. This study may provide novel insights into the function of Kir7.1.

### 4.1 Kir7.1 is expressed in the spinal cord neurons

Kir channels, which conduct atypical inward (rather than outward) K^+^ currents at depolarized membrane potentials, play key roles in ion homeostasis and neuronal excitability. There are seven subfamilies of Kir (Kir1‐7) discovered so far and each of which has multiple subfamily members.^33^ As one of the newest members of the Kir channel family, Kir7.1 has not been extensively studied in the central nervous system (CNS) and its function remains largely unknown, especially in pathological pain. Previous studies have been demonstrated that Kir7.1 is expressed in a variety of tissues and organs, such as the kidney,^34^ small intestine,^35^ gastric parietal cells, thyroid follicular cells, choroid plexus epithelium,^36^ retinal pigment epithelium,^37^ testis, liver, and prostate.^11^ In the CNS, it has been reported recently that Kir7.1 is widely expressed in the cortex, cerebellum, hippocampus, hypothalamus, pons, and striatum,^12^ while its distribution in the spinal cord is still unclear.

In the present study, we observed the expression of Kir7.1 in the spinal cord. Immunofluorescence results showed that Kir7.1 is expressed in the spinal cord, and predominantly co‐localized with NeuN, but not with GFAP or Iba1, indicating that Kir7.1 was expressed in the spinal neurons but not astrocytes or microglias in normal rats. We provided a piece of novel evidence for the wide distribution of Kir7.1 in CNS.

### 4.2 Kir7.1 contributes to the development of SNI‐induced chronic pain

Accumulating evidence has highlighted that K^+^ channels may play a crucial role in nociceptive processing through its dominant contributions in regulating neuron excitability**.** ^6^ Studies have suggested that changes in K^+^ channel expression may be important in the pathophysiology of peripheral sensitization and neuropathic pain.^10^, ^38^ The present work explored the role of Kir7.1 in the SNI‐induced mechanical allodynia. Western blotting assays showed that both the total and membrane expression of Kir7.1 in the spinal dorsal horn was significantly decreased by SNI surgery compared with the sham rats, indicating that the spinal Kir7.1 may be involved in the development of SNI‐induced chronic pain. Indeed, behavioral testing found that intraperitoneal injection of the Kir7.1 selective blocker ML418 induced mechanical allodynia behavior in naive rats, which was similar to the behavior in rats after SNI treatment. Moreover, we depleted Kir7.1 from WT rats by intrathecal injection of siRNA also induced the mechanical allodynia. These data suggest that Kir7.1 serves as an inhibitor for the genesis of neuropathic pain and downregulation of Kir7.1 in spinal neurons is essential for mechanical hypersensitivity induced by peripheral nerve injury.

It is Kir channel that is open in its steady state to create the resting membrane potential that most cells obey.^11^ The Kir7.1 channels provide a steady background K^+^ current to help set resting membrane potentials^11^ and appear to be essential for depolarization of the cells in which it is expressed.^39^ We assumed that the downregulation of kir7.1 might lead to the decrease in Kir7.1 current, which affects the resting potential and repolarization process of the membrane, thus increases the excitability and leads to pain hypersensitivity.

### 4.3 Kir7.1 SUMOylation is involved in the mechanism of SNI‐induced chronic pain

SUMOylation is a covalently reversible binding process between small ubiquitin‐like modifying proteins (SUMO1, 2, or 3) and the specific substrate. A variety of neuronal proteins have been identified as SUMO substrates, and disrupting the SUMO modification of these proteins results in defects in several kinds of neuronal function, including neuronal excitability.^19^, ^40^ Several K^+^ channels subunits are known to be SUMOylated, including K2P1,^41^ Kv1.5,^42^ Kv2.1,^23^, ^43^ Kv7,^44^ Kv4.2,^45^ and Kv11.1.^46^ Whether Kir7.1 can be SUMOylated has not been reported. The present work found that Kir7.1 channel was decorated by SUMO‐1 but not SUMO‐2/3 in the spinal dorsal horn of SNI rats. Treatment with GA (an E1 inhibitor) or 2‐D08 (a select UBC9 inhibitor) significantly decreased the appearance of SUMOylated Kir7.1 induced by SNI. These data suggested that Kir7.1 can be SUMOylated after SNI. To our knowledge, this is the first report that Kir7.1 can be post‐translationally modified by SUMO. Recent studies demonstrated that enhanced ion channel SUMOylation can contribute to hyperalgesia in models of chronic pain.^18^, ^47^ Therefore, we proposed that SUMO modification of Kir7.1 might contribute to SNI‐induced mechanical allodynia.

### 4.4 SUMOylation regulates surface expression of Kir7.1 channel at the plasma membrane

Channel activity and number of channels in the membrane are in part regulated by post‐translational modifications.^43^ Emerging evidence highlights that SUMO modification modulates the surface expression and biophysical properties of ion channels. It was reported that stable overexpression of Ubc9 in skeletal muscle cells resulted in reduced expression of the glucose transporters 1 (GLUT1), while increased expression of GLUT4.^48^ Subsequent investigations indicated more membrane protein expression regulated by SUMOylation, but not consistent. For example, SUMOylation lead to elevation of channel density at the cell surface, such as TRPM4,^24^ Kv4.2, ^45^ and VEGFR2,^49^ while unchanged in KV11.1.^46^ Taken together, the above evidence strongly indicated that the effect of SUMOylation on protein surface expression is substrate specificity. Our present work found that the membrane expression of Kir7.1 in the spinal dorsal horn was significantly decreased after SNI. Moreover, inhibiting SUMOylation by GA or 2‐D08 significantly increased the spinal membrane Kir7.1, suggesting that SUMOylation of Kir7.1 play a critical role in regulating its membrane expression in spinal cord neurons.

The regulation by SUMO has been shown to control membrane trafficking. SUMOylation retains VEGFR2 in the Golgi, blocking it trafficking to cell surface, reduces its surface expression, and attenuating VEGFR2‐dependent signaling in mice.^49^ Upon kainite or glutamate‐induced stimulation, GluR6 is SUMOylated and subsequently internalized, lead to a downregulation in GluR6 expression and a decrease in KAR‐EPSC amplitude.^22^ We assumed that SNI‐induced decrease in Kir7.1 surface expression might be due to SUMO‐modification controlling membrane trafficking and/or endocytosis of the channels. The detailed mechanism requires further studies.

Besides regulating the surface expression, SUMO pathway also operates directly at the plasma membrane and to control ion channel function.^41^, ^43^, ^44^, ^45^, ^46^, ^50^ With the existing data generally suggesting that enhanced SUMOylation of K^+^ channel subunits inhibit outward currents and increase cell excitability,^42^, ^43^, ^44^, ^45^, ^46^ we speculated that the SUMOylation of Kir7.1 have the potential to increase the excitability of neurons by altering K^+^ current through the channel and modulating either the action potential duration or resting membrane potential. Future studies are needed to confirm it.

## 5 CONCLUSION

In summary, the present study provided the first evidence that Kir7.1 is expressed in spinal cord neurons and Kir7.1 can be SUMOylated with SUMO1. SUMOylation of Kir7.1 might contribute to the development of SNI‐induced mechanical allodynia by decreased Kir7.1 surface expression. Our findings suggest that modulation of Kir7.1 function or selectively reversing SUMOylation of Kir7.1 is a potential target for neuropathic pain therapeutics.

## CONFLICT OF INTEREST

The authors declare no conflict of interest.

## DISCLOSURE

This study has never been published elsewhere.

## Supporting information


Figure S1

## Data Availability

The data that support the findings of this study are available from the corresponding author upon reasonable request.
