# Supplementary material for: SUMOylation of Kir7.1 participates in neuropathic pain through regulating its membrane expression in spinal cord neurons
Source: CNS Neurosci Ther. 2022 May 27;28(8):1259–67. doi: 10.1111/cns.13871 (PMC9253747; doi:10.1111/cns.13871)
Supplement: Supplementary file 1 — Figure S1 [file CNS-28-1259-s001.docx]

**
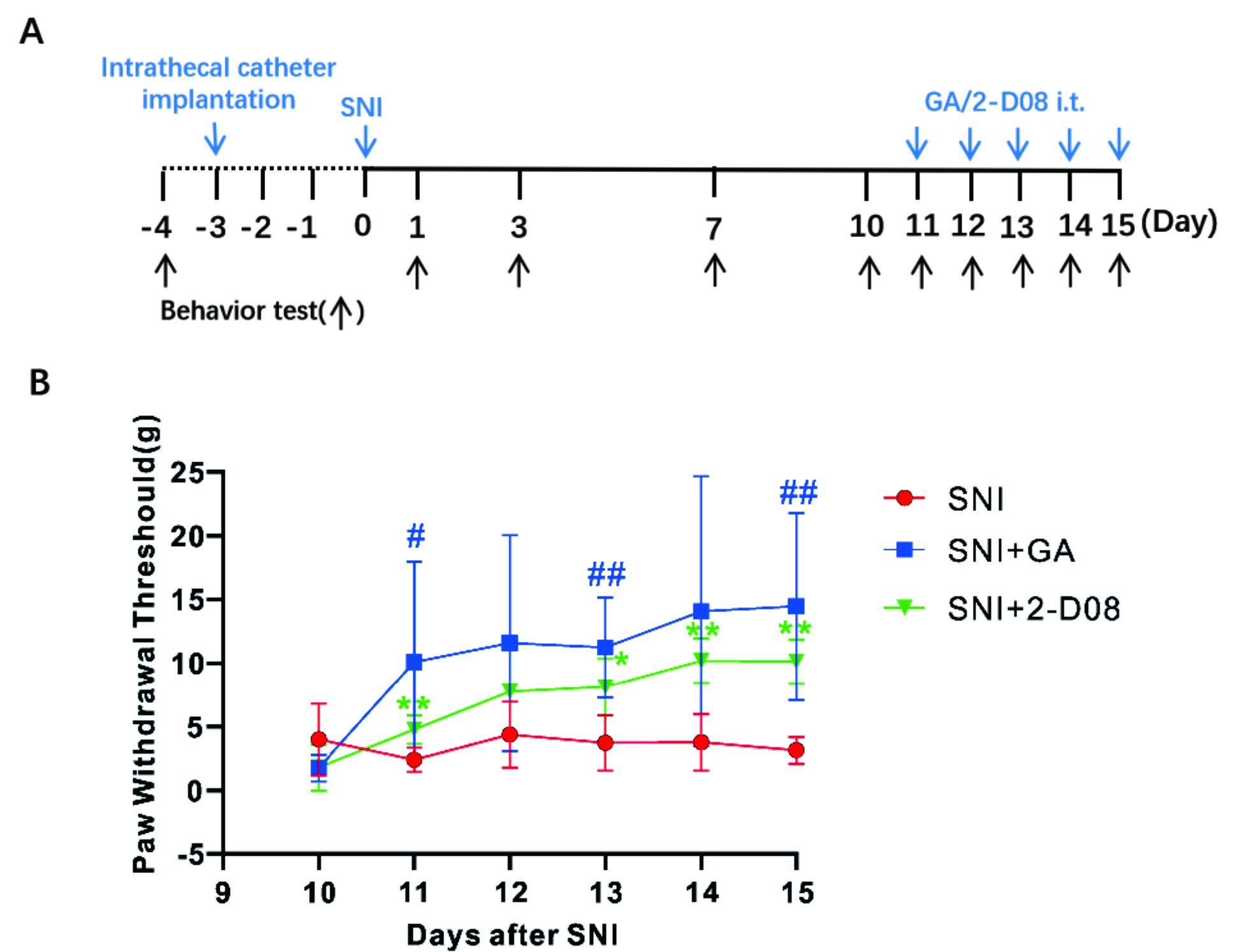
**

**Supplementary figure. Effects of SUMOylation inhibitor (GA or 2-D08)** **on SNI-induced mechanical allodynia in rats. (**A) Schematic of the behavior test and GA or 2-D08 injection experiments. (B) Intrathecal administration of GA (100 μM) or 2-D08 (60 μM) for 5 consecutive days from day 11 after SNI treatment significantly alleviated SNI-induced mechanical allodynia (n = 7 in SNI+GA group, # *P* < 0.05 versus the SNI group, ## *P* < 0.01 versus the SNI group, n = 4 in SNI+2-D08 group, * *P* < 0.05 versus the SNI group, ** *P* < 0.01 versus the SNI group).
